# LONG-TERM MEDICAL AND PSYCHOSOCIAL VULNERABILITY AFTER HOME RETURN FOLLOWING SEVERE TRAUMATIC BRAIN INJURY

**DOI:** 10.2340/jrm.v58.46093

**Published:** 2026-07-23

**Authors:** Andrea CALDERONE, Carmela RIFICI, Donatella BONAIUTI, Alfredo MANULI, Daniele BRUSCHETTA, Marco PICCIONE, Tina BALLETTA, Angelo QUARTARONE, Rocco Salvatore CALABRÒ

**Affiliations:** 1 IRCCS Centro Neurolesi Bonino-Pulejo, Messina; 2 Golgi Redaelli Institute, Milan; 3 Department of Biomedical, Dental Sciences and Morphological and Functional Images, University of Messina, Messina, Italy

**Keywords:** brain injuries, traumatic, rehabilitation, longitudinal studies, community participation, quality of life, patient-reported outcome measures, latent class analysis

## Abstract

**Objective:**

To identify a 5-year medical and psychosocial vulnerability profile among adults living at home 1 year after severe TBI and examine associated early characteristics.

**Design:**

Retrospective secondary analysis of a multicentre longitudinal cohort.

**Subjects/Patients:**

Adults aged 16 years or older with operationally defined severe traumatic brain injury, private residence at 1 year, linked 5-year follow-up, and complete data for 4 profile indicators.

**Methods:**

Latent class analysis used 5-year rehospitalization, PHQ-9, GAD-7, and Satisfaction With Life Scale scores dichotomized with prespecified clinically interpretable thresholds. External rehabilitation outcomes and early associated factors were examined descriptively and with multivariable logistic regression.

**Results:**

Among 2,835 participants, a 2-class solution identified a lower-vulnerability profile (*n* = 2,361) and a multidomain vulnerability profile (*n* = 474). The latter showed more depressive symptoms, anxiety symptoms, low life satisfaction, and rehospitalization, with poorer 5-year functioning, participation, health, productive status, and higher frequency of non-private residence. Better 1-year global outcome was protective; female sex, preinjury illicit drug use, and living alone at 1 year were associated with assignment to this profile.

**Conclusion:**

Home return after severe traumatic brain injury should be treated as a transition point for longitudinal rehabilitation surveillance.

## LAY ABSTRACT

Returning home after a severe traumatic brain injury is an important step, but it does not always mean that recovery is stable. This study looked at adults who were living at home 1 year after injury and followed their situation 5 years after injury. A subgroup had several later difficulties at the same time, including low mood, anxiety, low satisfaction with life, and further hospital care. This group also had poorer daily functioning, lower participation in community life, poorer general health, lower productive activity, and less stable living arrangements. The findings suggest that people who return home after severe traumatic brain injury may still need planned follow-up and support over time. Rehabilitation services should view home return as a point for continued monitoring, not as the end of recovery.

Severe traumatic brain injury (TBI) is increasingly understood as a condition with long-term rehabilitation consequences rather than as a time-limited acute event. Survival, medical stabilization, and discharge destination are important early milestones, but they do not fully describe the later trajectory of functioning, participation, and well-being. The chronic-disease framing of TBI is especially relevant for rehabilitation medicine, because recovery after severe injury often unfolds across multiple domains and may be shaped by personal, social, and environmental factors long after the inpatient episode has ended (1–3).

The International Classification of Functioning, Disability, and Health helps frame recovery after TBI as more than the reduction of impairment. It links body functions, daily activities, participation, environmental context, and health-related needs, which is directly relevant to long-term rehabilitation planning (4). This perspective is consistent with rehabilitation outcome recommendations for moderate to severe TBI and with work showing that participation measures after TBI map to multiple International Classification of Functioning, Disability, and Health categories (5, 6). A patient may return to a private residence and still experience restrictions in community participation, productive roles, emotional well-being, life satisfaction, or recurrent medical need. Home return is therefore clinically meaningful, but it remains a low-resolution marker of durable recovery.

Previous rehabilitation studies have shown that community integration and participation after moderate to severe TBI change over time and are lower among people with poorer functional recovery, greater psychosocial burden, and reduced social resources (7–10). Life satisfaction and vocational participation may also remain affected many years after injury, even among people living in the community (11, 12). Rehospitalization is another important signal because it reflects later medical instability and has been linked with participation after TBI (13, 14). Longitudinal work in the Traumatic Brain Injury Model Systems (TBIMS) has also shown that functional trajectories after inpatient rehabilitation are heterogeneous (15). These domains are usually analysed separately, yet they may cluster in ways that are more clinically informative than a single endpoint.

A multidomain approach is especially important in people who appear to have achieved a favourable residential milestone. Adults living in a private residence 1 year after severe TBI may be considered to have crossed a threshold of successful community return. However, that status may obscure later heterogeneity in functioning, participation, psychological health, life satisfaction, and health service use. Identifying a profile of later vulnerability within this apparently successful subgroup may help rehabilitation clinicians interpret home return more cautiously and plan surveillance beyond the early post-acute period.

Latent class analysis is useful for this purpose because it can describe patterns of co-occurring adverse indicators without assuming that every individual has the same combination of problems. In the present context, the method was used as a descriptive tool to summarize observed heterogeneity across rehabilitation-relevant domains. The resulting profile should therefore be understood as a probabilistic, data-derived pattern that can inform interpretation of long-term outcomes, not as a diagnostic category or biological subtype.

The aim of this study was to identify whether a distinct 5-year multidomain vulnerability profile could be identified among adults with severe TBI who were living in a private residence 1 year after injury, and to examine early demographic, preinjury, injury-related, rehabilitation, and 1-year characteristics associated with that profile. The prespecified hypothesis was that a clinically coherent subgroup would show later multidomain vulnerability despite 1-year home residence, and that lower preinjury resources, reduced social support, and poorer 1-year recovery would be associated with that profile.

## METHODS

### Study design, data source, and ethics

This retrospective secondary analysis used the de-identified public-use TBIMS longitudinal cohort. The TBIMS is a multicentre United States programme that follows people who sustain moderate to severe traumatic brain injury, receive acute hospital care, and complete inpatient rehabilitation. Baseline and rehabilitation variables were obtained from Form 1, and follow-up variables from Form 2. The primary outcome was assessed at the 5-year follow-up. The public-use cohort and follow-up instruments have been described previously and are suitable for secondary outcomes research when missingness and public-use recoding are considered (16, 17). Because this study used a fully de-identified public-use dataset and involved no new participant contact or collection of identifiable information, local ethics committee approval was not required.

### Cohort selection

The baseline file contained 20,167 participant records. Adults were defined as participants aged 16 years or older at injury. Severe TBI was operationalized *a priori* using public-use markers available in Form 1: admission Glasgow Coma Scale total score of 8 or lower after exclusion of noninformative codes, post-traumatic amnesia duration greater than 7 days or persistent post-traumatic amnesia at rehabilitation discharge, and/or craniotomy or craniectomy for intracranial pathology. Participants meeting at least 1 criterion were classified as having severe traumatic brain injury. This pragmatic definition should be interpreted as an operational cohort definition rather than as a single uniform biological state. It was selected to balance clinical severity with the constraints of de-identified public-use variables and to avoid relying on a single acute marker that might be missing, confounded by sedation or affected by local documentation practices.

**Fig. 1:**
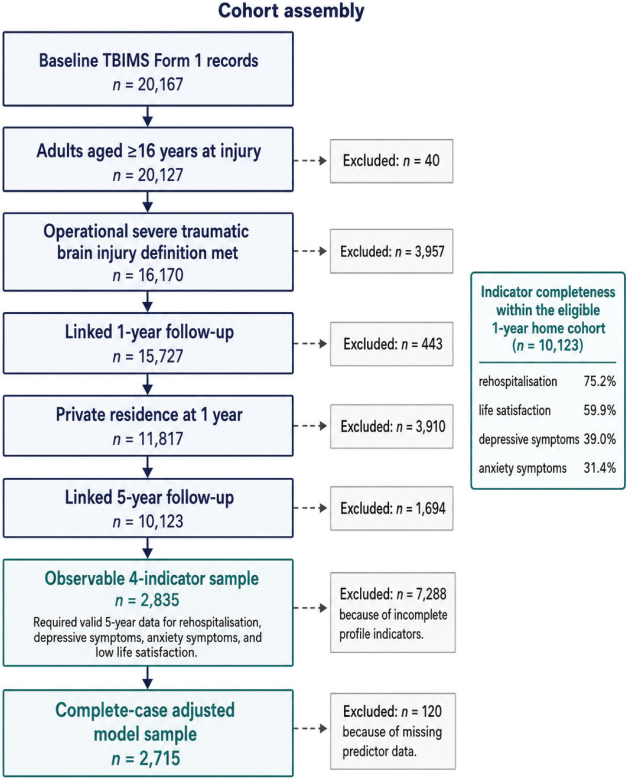
Cohort assembly for the 1-year home-dwelling severe traumatic brain injury sample and 5-year analytic cohorts. The primary latent profile analysis required valid 5-year data for all 4 defining indicators. The complete-case adjusted model further excluded participants with missing predictor data.

Baseline records were linked to follow-up records by study identifier. The cohort was restricted sequentially to participants with a linked 1-year follow-up record, those living in a private residence at 1 year, and those with a linked 5-year follow-up record. Private residence was defined by the home-based residence code in Form 2. Participants in non-private settings or with unknown residence status at 1 year were excluded from the home-dwelling cohort. The primary latent profile analysis required valid 5-year data on all 4 defining indicators after exclusion of structural and noninformative codes. This yielded an observable 4-indicator sample of 2,835 participants. The primary adjusted regression model further excluded participants with missing predictor data, yielding a complete-case analytic sample of 2,715 participants. Cohort assembly is shown in Fig. 1.

Public-use TBIMS variables include valid response codes as well as structural or noninformative codes, including not applicable, variable did not exist, no data from the person with TBI, refused, and unknown. Structural and noninformative codes were treated as missing when a variable was required for eligibility, profile definition, or adjusted modelling. The 4 class-defining indicators were not imputed because the latent classes were intended to describe observed co-occurrence of clinically interpretable states. Imputation of affective symptoms and life satisfaction in severe TBI would have required strong assumptions concerning data being missing at random, despite the possibility that missing self-report data reflected cognitive, communication, or clinical limitations. Consequently, assigned class frequencies describe the observable 4-indicator sample and should not be interpreted as prevalence estimates for the full eligible 1-year home-dwelling cohort.

### Outcome definition and rehabilitation framework

The primary outcome was assignment to a 5-year multidomain vulnerability profile among participants living in a private residence at 1 year. Four indicators were chosen to capture different clinically recognizable long-term problems after home return: any rehospitalization in the prior year, depressive symptoms, anxiety symptoms, and life satisfaction. Rehospitalization represented recurrent health-service need or medical instability; Patient Health Questionnaire-9 and Generalized Anxiety Disorder-7 scores represented depressive and anxiety symptom burden; and the Satisfaction With Life Scale represented subjective well-being. In an International Classification of Functioning, Disability, and Health-informed interpretation, these indicators span body functions, perceived well-being, and health-service use, while the external validators described below were used to examine activities, participation, and social-context relevance. Depressive symptoms were defined as Patient Health Questionnaire-9 score of 10 or greater, anxiety symptoms as Generalized Anxiety Disorder-7 score of 10 or greater, and low life satisfaction as Satisfaction With Life Scale score of 14 or lower (18–20). Rehospitalization was defined as any hospitalization in the prior year at the 5-year follow-up.

The thresholds were prespecified and were retained as binary indicators to make the latent profile clinically interpretable across domains with different scale ranges. Patient Health Questionnaire-9 and Generalized Anxiety Disorder-7 thresholds of 10 or greater are commonly used to identify at least moderate depressive or anxiety symptom burden, and Satisfaction With Life Scale scores of 14 or lower indicate low life satisfaction (18–20). This approach was intended to identify domains likely to be actionable during rehabilitation follow-up, not to imply that all indicators had equal severity or clinical weight. Continuous symptom and life-satisfaction distributions were examined descriptively to support interpretation of the dichotomized indicators.

To examine whether the latent profile reflected broader rehabilitation status, profiles were compared on outcomes that were not used to construct the latent classes. These external validators included 5-year Glasgow Outcome Scale–Extended score, Participation Assessment With Recombined Tools–Objective summary score, self-rated general health, productive status, and residence at 5 years (21, 22). Productive status followed the prespecified analytic grouping of competitive or special employment, student status, volunteer work, and leave from work without pay vs all other valid categories.

### Candidate predictors

Candidate predictors were selected *a priori* based on temporal precedence, clinical plausibility, and availability in the public-use dataset. The primary adjusted model retained age at injury, sex, race and ethnicity, years of education, productive preinjury status, illicit drug use in the year before injury, post-traumatic amnesia category, rehabilitation length of stay, 1-year Glasgow Outcome Scale–Extended score, and living alone at 1 year. The dataset provided sex as a binary variable; gender identity was not available. Race and ethnicity were represented as White non-Hispanic vs non-White race or Hispanic ethnicity in the primary model to balance parsimony and cell stability. Productive preinjury status used the same grouping applied to the 5-year productive status variable.

### Statistical analysis

Continuous variables were summarized with mean (standard deviation) or median (interquartile range), as appropriate. Categorical variables were summarized as counts and percentages. Between-profile comparisons were examined using χ^2^ or Fisher’s exact tests for categorical variables and Welch tests or Mann–Whitney *U* tests for continuous variables, according to distribution and scale. These comparisons were descriptive and were not interpreted causally.

Latent class analysis was fitted to the 4 binary outcome indicators in the observable 4-indicator sample. One-class to 4-class solutions were compared using the Akaike information criterion, Bayesian information criterion, sample-size-adjusted Bayesian information criterion, entropy, mean posterior probabilities, smallest class size, and clinical interpretability. After model selection, participants were assigned to the class with the highest posterior probability. Assigned class frequencies were interpreted only within the observable 4-indicator sample and not as prevalence estimates for the full eligible home-dwelling cohort. The primary purpose of the latent model was descriptive classification of a long-term rehabilitation outcome pattern. No attempt was made to develop or validate a clinical prediction score, and the downstream regression analysis was used to examine explanatory associations with early characteristics.

Multivariable logistic regression was used to estimate associations between early characteristics and assignment to the multidomain vulnerability profile in the complete-case analytic sample. Predictors were retained based on pre-specification and clinical relevance rather than automated selection. Adjusted odds ratios, 95% confidence intervals, and 2-sided *p*-values were reported. Model fit was summarized with McFadden pseudo-R2. Sensitivity analyses examined an expanded predictor model, a 4-domain vulnerability-count outcome and an expanded 5-domain vulnerability-count outcome that incorporated fair or poor general health. Analyses were performed in Python using pandas, NumPy, statsmodels, SciPy, and openpyxl (https://www.python.org/). Reporting followed the Strengthening the Reporting of Observational Studies in Epidemiology guidance (23). Additional methodological details and supporting results, including cohort observability, indicator completeness, variable recoding, latent class model selection, item-response probabilities, continuous indicator distributions, extended 5-year validators, adverse-domain co-occurrence, sensitivity analyses, and adjusted-model observability and predictor completeness, are provided in Tables SI–SVII.

## RESULTS

### Cohort assembly and observability

Of 20,167 baseline records, 20,127 were adults at injury, 16,170 met the operational severe TBI definition, and 15,727 had a linked 1-year follow-up record. Among these participants, 11,817 were living in a private residence at 1 year and 10,123 also had a linked 5-year follow-up record. The observable 4-indicator sample included 2,835 participants, and the complete-case analytic sample included 2,715 participants. Indicator completeness within the 10,123-person eligible 1-year home cohort was uneven: valid 5-year data were available for rehospitalization in 75.2%, life satisfaction in 59.9%, depressive symptoms in 39.0%, and anxiety symptoms in 31.4%. Relative to the broader eligible home cohort, participants retained in the observable sample were somewhat younger, more educated, more often productive before injury, and less disabled at rehabilitation discharge and at 1 year. This pattern indicates selection towards participants with more complete follow-up and less severe observable disability.

### Baseline, injury, rehabilitation, and 1-year characteristics

Participants assigned to the multidomain vulnerability profile were more often female, more likely to identify as non-White race or Hispanic ethnicity, had fewer years of education, were less often married at injury, and were less often productive before injury than participants assigned to the lower-vulnerability profile. They also more often reported illicit drug use in the year before injury. Acute injury markers, including admission Glasgow Coma Scale score, post-traumatic amnesia category, and craniotomy or craniectomy, were broadly similar across profiles. The clearest early recovery difference was observed at 1 year: both groups had a median Glasgow Outcome Scale–Extended score of 6, but the multidomain vulnerability profile had a lower interquartile distribution (Table I).

**Table I:** Baseline, injury, rehabilitation, and 1-year characteristics according to 5-year multidomain vulnerability profile

Characteristic	Overall (*N* = 2,835)	Lower-vulnerability profile (*n* = 2,361)	Multidomain vulnerability profile (*n* = 474)	*p*-value
Age at injury, years, mean (SD)	37.1 (16.2)	37.4 (16.7)	35.5 (13.8)	0.007
Female sex, *n* (%)	747 (26.4)	594 (25.2)	153 (32.3)	0.002
Non-White race or Hispanic ethnicity, *n* (%)	892 (31.5)	712 (30.2)	180 (38.1)	< 0.001
Education, years, mean (SD)	12.9 (2.7)	13.1 (2.7)	12.1 (2.6)	< 0.001
Married at injury, *n* (%)	938 (33.1)	805 (34.1)	133 (28.1)	0.012
Productive preinjury status, *n* (%)	2,276 (80.4)	1,930 (81.8)	346 (73.0)	< 0.001
Illicit drug use in year before injury, *n* (%)	589 (20.9)	450 (19.2)	139 (29.6)	< 0.001
Admission Glasgow Coma Scale total, median (IQR)	8.0 (4.0–13.0)	8.0 (3.0–13.0)	8.0 (4.0–12.2)	0.922
Post-traumatic amnesia duration, *n* (%)				0.909
< = 7 days	237 (8.5)	201 (8.6)	36 (7.8)	
8–28 days	1,320 (47.4)	1,097 (47.2)	223 (48.5)	
> 28 days	884 (31.7)	741 (31.9)	143 (31.1)	
Still in post-traumatic amnesia on rehabilitation discharge	344 (12.4)	286 (12.3)	58 (12.6)	
Craniotomy/craniectomy, *n* (%)	831 (29.4)	685 (29.1)	146 (30.8)	0.485
Acute care length of stay, days, median (IQR)	18.0 (12.0–27.0)	18.0 (12.0–27.0)	19.0 (12.0–29.8)	0.122
Rehabilitation length of stay, days, median (IQR)	19.0 (12.0–31.0)	20.0 (13.0–32.0)	18.0 (12.0–29.0)	0.173
Discharge Functional Independence Measure total, mean (SD)	93.5 (18.1)	93.6 (18.2)	93.0 (17.9)	0.508
1-year Glasgow Outcome Scale-Extended, median (IQR)	6.0 (5.0–7.0)	6.0 (5.0–8.0)	6.0 (4.0–6.0)	< 0.001
Living alone at 1 year, *n* (%)	354 (12.5)	292 (12.4)	62 (13.1)	0.732

Baseline and early recovery characteristics differed mainly in sociodemographic and 1-year global outcome domains.

Values are *n* (%) unless otherwise indicated. Percentages use non-missing denominators. *p*-values were derived from χ^2^ or Fisher’s exact tests for categorical variables and Welch tests or Mann–Whitney *U* tests for continuous variables, as appropriate. Productive preinjury status followed the prespecified analytic grouping used in this study. Glasgow Coma Scale available-case denominators exclude noninformative codes. Post-traumatic amnesia categories use cleaned values, with persistent post-traumatic amnesia on rehabilitation discharge retained as a category and unknown values treated as missing.

### Latent profile solution

Latent class analysis supported a 2-class solution. Compared with the 1-class model, the 2-class solution showed substantially better fit, with Bayesian information criterion 9,695.75 and entropy 0.827. Three-class and 4-class solutions offered weaker separation without clinically useful improvement. Within the observable 4-indicator sample, the retained solution identified a lower-vulnerability profile comprising 2,361 participants (83.3%) and a multidomain vulnerability profile comprising 474 participants (16.7%). Mean posterior probabilities were 0.956 for the lower-vulnerability profile and 0.939 for the multidomain vulnerability profile.

**Fig. 2:**
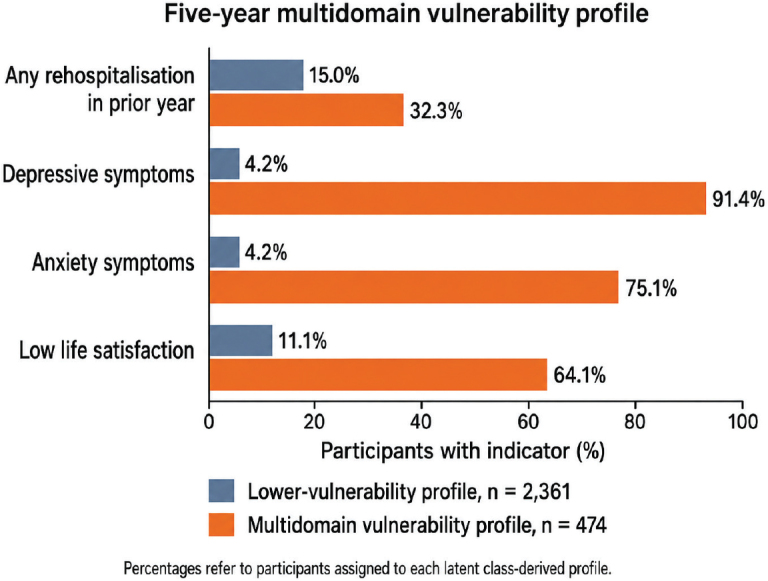
Five-year medical and psychosocial vulnerability profile after 1-year home return. Bars show the percentage of participants assigned to each latent class-derived profile who had each defining indicator at the 5-year follow-up. The vulnerability profile was characterized predominantly by depressive symptoms, anxiety symptoms, and low life satisfaction, with an additional rehospitalization signal.

Overall prevalences of the 4 defining indicators were 17.9% for rehospitalization, 18.7% for depressive symptoms, 16.0% for anxiety symptoms, and 20.0% for low life satisfaction. Compared with the lower-vulnerability profile, the multidomain vulnerability profile had higher prevalences of depressive symptoms (91.4% vs 4.2%), anxiety symptoms (75.1% vs 4.2%), low life satisfaction (64.1% vs 11.1%), and rehospitalization (32.3% vs 15.0%) (Table II; Fig. 2). The profile was therefore driven most strongly by affective symptoms and low life satisfaction, with an additional, smaller signal of medical instability.

**Table II:** Five-year multidomain vulnerability indicators and selected external rehabilitation outcomes by profile

Characteristic	Overall (*N* = 2,835)	Lower-vulnerability profile (*n* = 2,361)	Multidomain vulnerability profile (*n* = 474)	*p*-value
Panel A. Profile-defining 5-year indicators				
Any rehospitalization in prior year, *n* (%)	508 (17.9)	355 (15.0)	153 (32.3)	< 0.001
Depressive symptoms, Patient Health Questionnaire-9 > = 10, *n* (%)	531 (18.7)	98 (4.2)	433 (91.4)	< 0.001
Anxiety symptoms, Generalized Anxiety Disorder-7 > = 10, *n* (%)	454 (16.0)	98 (4.2)	356 (75.1)	< 0.001
Low life satisfaction, Satisfaction With Life Scale < = 14, *n* (%)	567 (20.0)	263 (11.1)	304 (64.1)	< 0.001
Panel B. Selected 5-year external rehabilitation validators				
5-year Glasgow Outcome Scale-Extended, median (IQR)	6.0 (5.0–8.0)	7.0 (6.0–8.0)	5.0 (4.0–6.0)	< 0.001
Participation Assessment With Recombined Tools-Objective summary score, mean (SD)	1.9 (0.7)	1.9 (0.7)	1.5 (0.6)	< 0.001
Fair/poor general health, *n* (%)	404 (21.8)	235 (15.2)	169 (56.3)	< 0.001
Non-private residence at 5 years, *n* (%)	46 (1.6)	31 (1.3)	15 (3.2)	0.007
Productive status at 5 years, *n* (%)	1,382 (48.8)	1,232 (52.2)	150 (31.6)	< 0.001

The multidomain vulnerability profile showed higher adverse 5-year indicators and poorer external rehabilitation outcomes.

Values are *n* (%) unless otherwise indicated. Glasgow Outcome Scale–Extended is shown as median (IQR); Participation Assessment With Recombined Tools-Objective summary score is shown as mean (SD). Fair/poor general health was defined as self-rated health of fair or poor among participants with valid general health data. Productive status at 5 years followed the prespecified analytic grouping. For Panel B categorical outcomes, percentages were calculated using variable-specific non-missing denominators. *p*-values in Panel A are descriptive because the indicators were used to derive the latent profile.

### External rehabilitation validators

External validators supported the clinical coherence of the latent class-derived profile. Participants in the multidomain vulnerability profile had lower 5-year Glasgow Outcome Scale–Extended scores, lower Participation Assessment With Recombined Tools–Objective summary scores, poorer self-rated general health, lower productive status, and less stable residence at 5 years than participants in the lower-vulnerability profile. High global outcome, defined as Glasgow Outcome Scale–Extended score of 7 or 8, was achieved by 16.8% of the multidomain vulnerability profile compared with 52.0% of the lower-vulnerability profile. Productive status at 5 years was present in 31.6% of the multidomain vulnerability profile and 52.2% of the lower-vulnerability profile (see Table II).

### Multivariable associations

The primary adjusted model identified several early characteristics associated with assignment to the multidomain vulnerability profile. Better 1-year Glasgow Outcome Scale-Extended score was protective, with adjusted odds ratio 0.73 per 1-point increase (95% confidence interval 0.68–0.79; *p* < 0.001). Greater education (adjusted odds ratio 0.93 per year, 95% confidence interval 0.89–0.97; *p* < 0.001) and productive preinjury status (adjusted odds ratio 0.65, 95% confidence interval 0.50–0.85; *p* = 0.001) were also associated with lower odds. Female sex, preinjury illicit drug use, living alone at 1 year, and age 36–55 years were associated with higher odds of assignment to the multidomain vulnerability profile. Participants aged 56 years or older had lower odds than those aged 16–35 years. Race and ethnicity, post-traumatic amnesia category, and rehabilitation length of stay were not independently associated with assignment to the multidomain vulnerability profile in the primary model (Table III). Model-level fit was modest, with McFadden pseudo-R2 of 0.082, supporting interpretation as an explanatory association rather than a clinical prediction model.

**Table III:** Multivariable associations with the 5-year multidomain vulnerability profile

Predictor	Adjusted odds ratio	95% confidence interval	*p*-value
Age group			
16–35 years	Reference		
36–55 years	1.29	1.02–1.63	0.036
≥ 56 years	0.46	0.31–0.69	< 0.001
Female sex (vs male)	1.45	1.15–1.83	0.002
Non-White race or Hispanic ethnicity (vs White non-Hispanic)	1.15	0.91–1.44	0.240
Education, per 1 year	0.93	0.89–0.97	< 0.001
Productive preinjury status (vs non-productive)	0.65	0.50–0.85	0.001
Illicit drug use before injury (vs no)	1.52	1.19–1.94	< 0.001
Post-traumatic amnesia duration			
≤ 7 days	Reference		
8–28 days	1.08	0.72–1.63	0.713
> 28 days	0.87	0.56–1.35	0.533
Still in post-traumatic amnesia on rehabilitation discharge	0.77	0.46–1.27	0.300
Rehabilitation length of stay, per 10 days	0.95	0.89–1.00	0.059
1-year Glasgow Outcome Scale-Extended, per 1 point	0.73	0.68–0.79	< 0.001
Living alone at 1 year (vs with others)	1.39	1.01–1.91	0.044

Better 1-year global outcome was protective, while selected social-context and preinjury factors were associated with higher odds of later multidomain vulnerability.

Outcome = multidomain vulnerability profile vs lower-vulnerability profile. The complete-case regression sample included 2,715 participants, of whom 447 (16.5%) were assigned to the multidomain vulnerability profile. Odds ratios are adjusted for all variables shown and should be interpreted as explanatory associations rather than as a clinical prediction score. Reference categories were age 16–35 years, male sex, White non-Hispanic race/ethnicity, nonproductive preinjury status, post-traumatic amnesia < = 7 days, and not living alone at 1 year.

### Sensitivity analyses

Sensitivity analyses showed a consistent overall pattern. The expanded predictor model that added craniotomy or craniectomy and rehabilitation discharge Functional Independence Measure total score did not materially alter the primary associations. Alternative models based on 4-domain and 5-domain vulnerability-count outcomes produced similar associations for education, productive preinjury status, illicit drug use, and 1-year Glasgow Outcome Scale–Extended score. Associations for female sex, living alone, and older age were attenuated when fair or poor general health was incorporated into the broader vulnerability-count outcome.

## DISCUSSION

This study identified a clinically meaningful medical and psychosocial multidomain vulnerability profile among adults with severe TBI who were living in a private residence 1 year after injury. The profile was observed in 16.7% of the observable 4-indicator sample and was characterized by high levels of depressive symptoms, anxiety symptoms, and low life satisfaction, together with a smaller but meaningful rehospitalization signal. External validators showed poorer 5-year global functioning, participation, self-rated health, productive status, and residential stability. Early associated factors included 1-year global outcome, education, preinjury productive status, sex, preinjury illicit drug use, living alone at 1 year, and age group. These findings support the interpretation of home return as an important transition point rather than as a definitive marker of durable recovery.

The principal rehabilitation implication is that residential status alone is insufficient to characterize long-term recovery after severe traumatic brain injury. Previous studies have shown that community integration, participation and employment can evolve over several years, and that psychosocial factors often have major influence on later outcomes (7, 9, 10, 12). The present analysis extends that work by focusing on participants who had already achieved 1-year residence in a private home. Within this selected group, later vulnerability remained heterogeneous and clinically consequential. From an International Classification of Functioning, Disability, and Health perspective, the findings reinforce the need to examine functioning across linked domains rather than relying on a single global or residential endpoint.

The magnitude of the differences across external validators is clinically relevant. The contrast in high global outcome was large, and productive status was approximately 20 percentage points lower in the multidomain vulnerability profile. These differences are not merely statistical artefacts of a large cohort; they represent domains that affect independence, role fulfilment, caregiver demands, and service needs. The lower participation score and poorer self-rated health further suggest that the profile captured a broader rehabilitation state, even though participation and general health were not used to construct the latent classes. Direct neuropsychological measures were not part of the current profile, but cognitive deficits after severe TBI may influence participation, self-management, follow-up attendance, and the reliability of self-assessment. Future vulnerability profiles should therefore incorporate cognitive screening or neuropsychological assessment where available, together with communication status and proxy or caregiver perspectives when participant self-report is limited.

The profile structure also requires careful interpretation. The multidomain vulnerability profile was not driven equally by all 4 indicators. Separation was strongest for depressive symptoms, anxiety symptoms, and low life satisfaction, whereas rehospitalization was more modest. This does not weaken the clinical message. Rather, it suggests that later vulnerability after home return may be primarily psychosocial and well-being-related, accompanied in some individuals by recurrent medical need. Recent multidimensional participation and life-satisfaction studies in TBI similarly emphasize that participation and well-being are not captured by a single global endpoint (24–26). This interpretation is also consistent with evidence that emotional and social factors are closely related to participation after TBI and that life satisfaction can remain affected many years after injury (9, 11, 27–30).

The external validators strengthen confidence that the latent class-derived profile reflects more than measurement redundancy among affective and life-satisfaction indicators. Participants assigned to the multidomain vulnerability profile had poorer objective participation, lower productive engagement, poorer self-rated health, and lower global outcome. Productive status is particularly relevant in rehabilitation medicine because work, study, and structured productive roles are closely connected with participation, identity, and well-being. Long-term employment research after TBI has shown that vocational participation may continue to change over extended periods and that ongoing vocational rehabilitation may be beneficial for selected individuals (12). The present findings add that productive status is also embedded within a broader pattern of multidomain vulnerability.

The early association profile should be interpreted as explanatory, not predictive or causal. Better 1-year Glasgow Outcome Scale–Extended score was the strongest protective factor, suggesting that integrated early recovery remains clinically important even among people living at home. Education and preinjury productive status were also protective, while living alone at 1 year and preinjury illicit drug use were associated with higher odds of later vulnerability. These associations point towards the importance of social and contextual resources as well as injury-related recovery. The analysis included preinjury illicit drug use because it was temporally antecedent to the 5-year profile, but it could not distinguish new or recurrent post-injury addictions, changes in alcohol or drug use trajectories, pain-medication exposure, or behavioural-health treatment after injury. These factors should be examined in future longitudinal work. The association with female sex is consistent with the need to consider sex-specific patterns of post-traumatic symptoms and psychosocial burden, but the binary sex variable available in the public-use dataset did not allow analysis of gender identity or more detailed gendered social determinants.

The age pattern was less straightforward. Participants aged 36–55 years had higher odds of assignment to the multidomain vulnerability profile than those aged 16–35 years, whereas those aged 56 years or older had lower odds in the primary model. This finding should not be interpreted as evidence that older adults are intrinsically protected. The association was attenuated when general health was incorporated into a broader vulnerability-count outcome, and the observable sample may have selected older survivors who were healthy enough to complete follow-up indicators. Age-related findings may also be influenced by survivor bias, response shift, adaptation or accommodation to new functioning, differences in expectations regarding work and social roles, family support, and institutional residence pathways. For rehabilitation planning, age should not be used in isolation to infer later risk. A middle-aged person attempting to resume family and vocational roles may face a different vulnerability context from an older person with different role expectations, support structures, or service pathways.

**Fig. 3:**
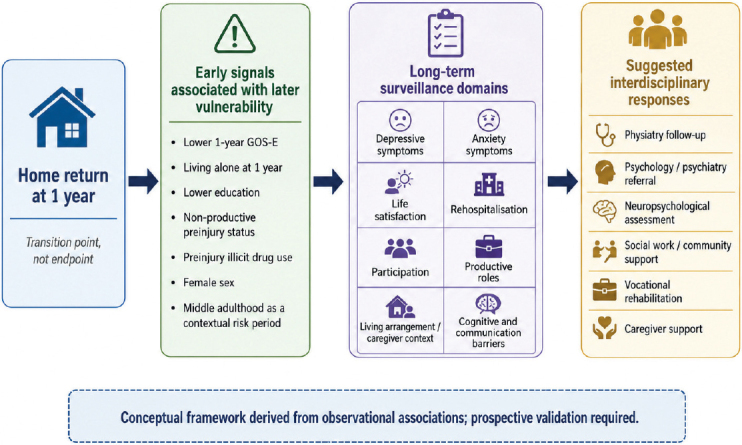
Conceptual surveillance framework after 1-year home return following severe traumatic brain injury. The figure summarizes clinically relevant early signals, follow-up domains, and interdisciplinary responses suggested by the present observational findings. It is intended as a conceptual framework and not as a validated clinical prediction pathway. GOS-E: Glasgow Outcome Scale–Extended.

The results do not support use of the profile as a stand-alone clinical screening instrument. The model was derived from 5-year indicators, and the regression model had modest explanatory fit. However, the findings do support more deliberate longitudinal surveillance after home return. These implications are summarized in a conceptual post-home-return surveillance framework (Fig. 3). In practice, home return after severe TBI could trigger structured follow-up that periodically reviews emotional symptoms, life satisfaction, rehospitalization, participation, productive roles, living arrangements, caregiver context, and cognitive or communication barriers to self-management. Screening tools such as the Patient Health Questionnaire-9, Generalized Anxiety Disorder-7 and Satisfaction With Life Scale, or clinically equivalent measures, could be combined with assessment of participation, role resumption, and recent health-service use. Findings should guide interdisciplinary referral pathways, including physiatry, psychology, neuropsychology, social work, vocational rehabilitation, and community-based services according to individual needs. Such surveillance should be tested prospectively before being implemented as a formal pathway.

A pragmatic surveillance model would not require all home-dwelling survivors to receive the same intensity of follow-up indefinitely. Rather, early recovery status and social-context signals may help clinicians decide who needs more structured review, supported referral pathways, or renewed interdisciplinary assessment. For example, people living alone, those with lower education, or those without productive preinjury roles may benefit from closer attention to environmental supports, community participation, and access to services. Evidence on community discharge, community integration, participation disparities, chronic pain, participation prediction, and physical therapy after TBI also supports the need for rehabilitation pathways that remain responsive beyond the early inpatient episode (31–37). These implications should be evaluated prospectively before being implemented as a formal pathway.

Future work should test whether similar profiles are found in cohorts with more complete affective, cognitive, and participation follow-up, in non-United States rehabilitation systems and in samples that include richer information on post-discharge care. Analyses that propagate latent class uncertainty, incorporate repeated measures over more than 2 follow-up waves, and examine service-use trajectories could clarify whether vulnerability emerges gradually, persists from earlier recovery, or fluctuates with changing health and social circumstances. Future profiles should also evaluate neuropsychological assessment, cognitive screening, communication status, caregiver-reported outcomes, and post-injury substance-use trajectories. Intervention studies could then evaluate whether structured follow-up reduces later multidomain burden.

Several strengths should be noted. The study used a large multicentre longitudinal cohort, restricted the analysis to a clinically important home-dwelling subgroup, integrated medical and psychosocial indicators, and examined external validators not used in profile derivation. The analysis also used prespecified predictors and sensitivity analyses, and reporting followed observational-study guidance. The study therefore provides a coherent rehabilitation outcomes perspective rather than a purely statistical classification exercise.

Limitations are also important. This was a secondary analysis of public-use data and is susceptible to residual confounding, information loss from public-use recoding, and unmeasured differences in rehabilitation access after discharge. Missing data were substantial. Complete data on all 4 defining indicators were available for only 2,835 of 10,123 otherwise eligible home-dwelling participants, with especially limited availability for affective measures. Class frequencies therefore describe the observable 4-indicator sample and should not be generalized as population prevalence estimates. Because participants retained in the observable sample were systematically somewhat younger, more educated, more often productive before injury, and less disabled, the 16.7% frequency of the multidomain vulnerability profile is likely a conservative underestimate of the true long-term burden among eligible home-dwelling survivors.

Several additional limitations relate to measurement. The complete-case regression model excluded an additional 120 participants because of predictor missingness. The profile relied partly on self-reported affective symptoms and life satisfaction, which may be difficult for people with severe TBI who have impaired insight, cognitive impairment, aphasia, or other communication limitations. Those individuals may have been less likely to complete Patient Health Questionnaire-9, Generalized Anxiety Disorder-7, or Satisfaction With Life Scale assessments and may therefore be underrepresented. Dichotomising Patient Health Questionnaire-9, Generalized Anxiety Disorder-7, and Satisfaction With Life Scale scores improved clinical interpretability but reduced information concerning symptom severity, variance, and dose-response gradients. The current profile also did not include direct neuropsychological assessment, post-injury substance-use trajectories, pain-medication exposure, or behavioural-health service use. The operational severe TBI definition was pragmatic and may have captured heterogeneous severity pathways. Downstream regression used assigned latent class membership rather than a method that explicitly propagated classification uncertainty, although entropy and posterior probabilities were good. Finally, all associations are observational and should not be interpreted causally.

In conclusion, among adults with severe TBI who were living in a private residence 1 year after injury, home return did not ensure durable recovery. A distinct 5-year multidomain vulnerability profile was identifiable and was associated with poorer functioning, participation, productive engagement, self-rated health, and residential stability. Community return after severe TBI should prompt longitudinal rehabilitation surveillance and interdisciplinary follow-up rather than be treated as the end of recovery.

## Supplementary Material


