# Supplementary material for: LONG-TERM MEDICAL AND PSYCHOSOCIAL VULNERABILITY AFTER HOME RETURN FOLLOWING SEVERE TRAUMATIC BRAIN INJURY
Source: J Rehabil Med. 2026 Jul 23;58:46093. doi: 10.2340/jrm.v58.46093 (PMC13403164; doi:10.2340/jrm.v58.46093)
Supplement: Supplementary file 1 [file JRM-58-46093-s1.pdf]

Supplementary tables provide cohort observability, indicator completeness, latent class model selection, profile probabilities, continuous score distributions and predictor-level missingness supporting the revised manuscript.

Table SI. Cohort assembly counts.

Step	n
Baseline TBIMS Form 1 records	20167
Adults aged >=16 years at injury	20127
Operational severe traumatic brain injury definition met	16170
Linked 1-year follow-up record	15727
Private residence at 1 year	11817
Linked 5-year follow-up record	10123
Observable 4-indicator sample	2835
Complete-case adjusted model sample	2715

Table SII. Completeness of 5-year profile-defining indicators within the eligible 1-year home cohort (N=10,123).

Indicator	Valid n (%)	Not valid n (%)
Any rehospitalisation in prior year	7617 (75.2)	2506 (24.8)
Depressive symptoms (PHQ-9 total)	3949 (39.0)	6174 (61.0)
Anxiety symptoms (GAD-7 total)	3183 (31.4)	6940 (68.6)
Life satisfaction (SWLS total)	6067 (59.9)	4056 (40.1)

Table SIII. Observable versus non-observable eligible participants.

Characteristic	Eligible home cohort (N=10,123)	Observable 4-indicator sample (n=2,835)	Not observable (n=7,288)
Age at injury, years, mean (SD)	38.0 (17.3)	37.1 (16.1)	38.4 (17.7)
Female sex, n (%)	2529 (25.0)	747 (26.3)	1782 (24.5)
Non-White race or Hispanic ethnicity, n (%)	3509 (34.7)	892 (31.5)	2617 (35.9)
Education, years, mean (SD)	12.5 (2.9)	12.9 (2.7)	12.3 (2.9)
Productive preinjury status, n (%)	7554 (74.6)	2276 (80.3)	5278 (72.4)
Discharge FIM total, mean (SD)	90.5 (21.4)	93.5 (18.1)	89.4 (22.4)
1-year GOS-E, median (IQR)	6.0 (4.0-7.0)	6.0 (5.0-7.0)	6.0 (4.0-7.0)

Table SIV. Latent class model selection.

Classes	Log_likelihood	AIC	BIC	ssaBIC	Entropy	Smallest_class_n	Smallest_class_pct
1	-5365.957	10739.91	10763.71	10751.0	None	2835	100.0
2	-4812.101	9642.2	9695.75	9667.15	0.827	474	16.7
3	-4809.409	9646.82	9730.12	9685.63	0.631	315	11.1
4	-4809.126	9656.25	9769.3	9708.93	0.633	101	3.6

Table SV. Class-specific item-response probabilities and assigned-class prevalences.

Indicator	LCA probability lower	LCA probability vulnerability	Assigned prevalence lower	Assigned prevalence vulnerability
Any rehospitalisation	0.152	0.291	0.15	0.323
Depressive symptoms	0.031	0.838	0.042	0.914
Anxiety symptoms	0.036	0.678	0.042	0.751
Low life satisfaction	0.116	0.552	0.111	0.641

Table SVI. Continuous PHQ-9, GAD-7 and SWLS scores by assigned profile.

Measure	Lower-vulnerability profile mean (SD)	Lower-vulnerability profile median (IQR)	Multidomain vulnerability profile mean (SD)	Multidomain vulnerability profile median (IQR)
PHQ-9 total score	2.2 (3.5)	0.0 (0.0-4.0)	14.4 (5.4)	14.0 (11.0-18.0)
GAD-7 total score	2.1 (3.5)	0.0 (0.0-3.0)	12.2 (5.5)	12.0 (10.0-16.0)
SWLS total score	24.2 (7.1)	25.0 (19.0-30.0)	13.8 (7.1)	12.0 (9.0-18.0)

Table SVII. Adjusted-model observability and predictor completeness.

Quantity or predictor	Valid n	Missing n	Interpretation
Observable 4-indicator sample	2,835	-	Valid 5-year data for all 4 profile-defining indicators
Complete-case adjusted model sample	2,715	120	Valid data for all prespecified model predictors
Outcome events in complete-case model	447	-	Assigned to the multidomain vulnerability profile
Age group	2,835	0	Derived from public-use age at injury
Sex	2,834	1	Valid male/female public-use code
Race/ethnicity grouping	2,831	4	White non-Hispanic vs non-White race or Hispanic ethnicity; participants with noninformative race/ethnicity coding were excluded from the complete-case model
Education	2,831	4	Valid coded years of education
Productive preinjury status	2,832	3	Valid calculated employment grouping
Illicit drug use before injury	2,819	16	Valid yes/no code
Post-traumatic amnesia category	2,785	50	Valid duration category or still in PTA at rehabilitation discharge
Rehabilitation length of stay	2,835	0	Valid continuous value
1-year Glasgow Outcome Scale-Extended	2,790	45	Valid 1-year GOS-E score
Living alone at 1 year	2,832	3	Valid 1-year living-with code

Note: Predictor-level counts do not sum to the complete-case exclusion count because missingness can overlap across predictors. The adjusted model was interpreted as an explanatory association model, not as a clinical prediction score.

Abbreviations

AIC, Akaike information criterion; BIC, Bayesian information criterion; FIM, Functional Independence Measure; GAD-7, Generalized Anxiety Disorder-7; GOS-E, Glasgow Outcome Scale-Extended; IQR, interquartile range; LCA, latent class analysis; PHQ-9, Patient Health Questionnaire-9; PTA, post-traumatic amnesia; SD, standard deviation; ssaBIC, sample-size-adjusted Bayesian information criterion; SWLS, Satisfaction With Life Scale; TBI, traumatic brain injury; TBIMS, Traumatic Brain Injury Model Systems.
